# Effect of *Rehmannia glutinosa* Libosch extract on proliferation and cardiogenic pre-differentiation of human mesenchymal stem cells

**DOI:** 10.37796/2211-8039.1243

**Published:** 2022-03-01

**Authors:** Huu Dat Nguyen, Len Ho Thi, Xuan Bach Ho, Van Anh Cao, Duy Minh Le Hoang

**Affiliations:** College of Sciences, Hue University, Viet Nam

**Keywords:** *Rehmannia glutinosa* extract, Mesenchymal stem cells, Proliferation, Cardiogenic differentiation

## Abstract

**Background:**

Vietnamese medicine tried and tested certain bioactive compounds from plants to increase the rate of tissue immunomodulation, regeneration, and differentiation. Although there are many research papers discovered about phytochemicals of *Rehmannia glutinosa* Libosch and differentiation induction potential of some substances purified from this herbal, it finds difficult to seek research that investigated the effect of hot water-extracted *R. glutinosa* Libosch (RGE) on proliferation and cardiogenic differentiation of mesenchymal stem cells, even though it has commonly been used for a long time because of its function as a restorative and as a critical role in cardiovascular treatment in traditional.

**Results:**

Our research indicated that RGE has many predicted bio-pharmacological effects, and the RGE is demonstrated that it is non-toxic to UC-MSCs (IC50 = 1274 ppm). It also stimulates the proliferation and migration of UC-MSCs at various concentrations, especially at the RGE concentration of 50 ppm, during four days of treatment. On the other hand, the RGE can induce the cardiac pre-differentiation process from the fifth day to the fifteenth day after treatment, which was proven through both molecular and cellular (morphology evidence) levels like the up-regulation of GATA4, Nkx2.5, cTnT α-MHC, Desmin genes; the expression of Desmin protein, the appearance of two-nuclei cells, connecting process of adjoining cells, the cytoplasmic striations.

**Conclusion:**

The RGE could either stimulate proliferation–migration of MSCs or induce the cardiac pre-differentiation process. This extract can be classified as non-toxic to the UC-MSCs.

## 1. Introduction

*Rehmannia glutinosa* Libosch (RG) is a carbohydrate-rich plant in the family of Scrophulariaceae and is a famous folkloric medicinal plant in Vietnam. Research works in the past revealed that RG contains many components in its extract [1], which is responsible for various pharmacological effects such as treatment potential on osteoporosis [2], hypoglycemia in various diabetic disorders [3], anti-senescence [4], cardiovascular diseases [5], heart failure [6], and tumor [7]. We can easily search for research regarding RG's phytochemical screening or the effect of some components purified from RG on the differentiation of stem cells [8,9]; and on inhibiting cancer cells [7]; on neuroprotection [10]. There is a lack of information and knowledge about the impact of hot water-extracted *R. glutinosa* Libosch on mesenchymal stem cells even though residents of Eastern countries often use RG with a form like crude extract instead of using the purified individual components. Moreover, there is a tendency, which is more and more popular in research, to use the plant extract for proliferating and differentiating on the MSCs with the various promising outputs from many different plants such as *Glycyrrhiza glabra, Rhizoma Drynariae, Foeniculum vulgare* [11], so the use of RG's extract is a good goal at the moment.

Besides, although the use of bone marrow-derived mesenchymal stem cells (BMSCs) has been popular in research and clinical studies, umbilical cord mesenchymal stem cells (UC-MSCs) can become an alternative source to BMSCs due to non-invasive collection protocol and easy accessibility. They have also emerged strongly to the attractive therapy for many diseases because of their abundant sources, self-renewal ability, immunoregulation ability, differentiation into different kinds of mature cells or functional cells, and even non-mesodermal cell types either *in vitro* or *in vivo*. In this day and age, they have been used for various clinical trials for dealing with many kinds of sickness, especially the MSCs use for treating COVID-19 recently [12–14]. Since all of the advantages of UC-MSCs, it is clear that the search for UC-MSCs proliferation strategies is always necessary, especially for increasing the benefits of UC-MSCs on stem cell transplantation therapy. Moreover, RG's use for UC-MSCs proliferation stimulation is sensible when both support different diseases treatment.

Cardiovascular disease-related mortality accounts for the most significant percentage of deaths world-wide. The critical reason for this situation is that cardiomyocytes' s insufficient capacity to proliferate and regenerate themselves. As a result, following a cardiac attack, these resident cells are replaced by fibroblasts and non-contractile scar tissue, resulting in heart muscle tissue contractile dysfunction and heart failure. A variety of techniques are used to address this issue, including allogeneic heart transplantation and stem cell transplantation. However, stem cell transplantation therapy has certain drawbacks, such as organ trapping and spontaneous differentiation in *the vivo* microenvironment when using MSCs or tumor-forming when embryonic stem cells are used [15]. With the same treatment purpose and elimination of some above obstacles, the differentiated cells are the promising alternative sources to the above-mentioned therapy. Thus most scientists have discovered differentiating procedures into functional cardiomyocytes or cardiomyocytes-like cells *in vitro* from stem cells in the last decades, despite the differentiation into function cardiomyocyte from MSCs is controversial due to the failure in the majority of researches in the past, especially when scientists use herbal plant for differentiation purpose [16,17].However, this controversy is not very important since another publication reported the preferred appropriate cardiomyocytes-like cells for transplantation [17]. Thus, this scientific work would be intriguing and surprising if we combine the advantages of stem cells and RGE to induce the pre-differentiation into cardiomyocytes - like cells.

## 2. Method

### 2.1. Collection of R. glutinosa Libosch (RG) materials

The roots of RG were collected between January and February 2020 from Thanh Hoa, Vietnam. The sample was identified by Dr. Nguyen Viet Thang (Hue University), and the voucher specimen is deposited in the Sub-department of Applied Biology, Department of Biology, College of Sciences, Hue University (voucher number – D06).

### 2.2. Preparation of RGE

**Fig. 1:**
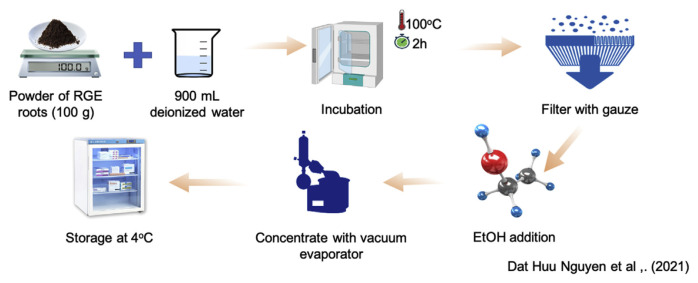
Protocol of hot water-extracted Rehmannia glutinosa Libosch extraction process.

The RGE was carried out using Jane C-J Chao's procedure [7] was modified for suiting to laboratory conditions. The fresh roots were dried at room temperature before grinding, and afterward, powder of RG root (100 g) was incubated with 900 mL deionized water at 100 °C for 2 h. The herbal juice was filtered with gauze. The filtered supernatant was precipitated with three volumes of 950 mL/L ethanol, concentrated by incubating in the water bath at 60 °C. The RGE was obtained and stored at 4 °C (Fig. 1).

### 2.3. Estimating the carbohydrate content of RGE by the phenol-sulfuric acid method

The total carbohydrate content in RGE was determined by a phenol-sulfuric acid assay using glucose as a standard.

Briefly, 100 mg RG powder and 5 mL of 2.5 N HCl were boiled in a water bath for 3 h for hydrolyzing, followed by cooling. After solid sodium carbonate was added for neutral, the solution's volume was made up to 100 mL and centrifugation. Three experiment tubes were set by 0.1 mL sample and 0.9 mL water; 0.2 sample and 0.8 mL water; 1 mL water (blank). Next, 5 mL of 96% sulphuric acid was added to each tube and shaken after 10 min. After that, these tubes were incubated at 25–30 °C for 20 min, and the colors were read at 490 nm. Finally, the sample's total carbohydrate content was calculated using the standard graph [18,19].

### 2.4. Composition analysis of RGE by GC–MS method

The RGE after extraction and storage in the above condition was split into 3 mL in a tube for GC–MS analysis.

The RGE was tested by the drug, cosmetic, and food quality control center of Thua Thien Hue province (HueQC). The composition was analyzed by the Gas Chromatography-Mass Spectrometry method (GC–MS).

### 2.5. Isolation and in vitro culture of human MSCs from the umbilical cord (UC-MSCs)

Umbilical cords were collected from Hue Central Hospital with the approval of the Research Ethics Committee of Hue Central Hospital and transported to the Stem cells Laboratory, Department of Biology, College of Sciences, Hue University in 0.9% normal saline containing 100 U/mL penicillin and 100 mg/mL streptomycin at 4 °C with written consents of mothers and families.

Initially, blood vessels were removed in saline, and the umbilical cords were sliced into 1–2 cm^2^ sections. These fractions were rinsed with PBS and subsequently suspended in StemMACS™ MSC Expansion Media Kit XF (Miltenyi Biotec, Germany). Incubation was performed at 37 °C in a humidified atmosphere containing 5% CO_2_, and the medium was replaced every three days. The morphology of UC-MSCs was followed with Olympus CKX31SF inverted microscope (Olympus Corporation, Tokyo, Japan).

When the cell population's confluence reached around 80%, the cells were trypsinized, counted, and re-seeded into culture dishes at a density of approximately 1000 cells/cm^2^.

### 2.6. Colony-forming unit-fibroblast (CFU–F) assay

For CFU-F culturing at passage 2–3, the cells were seeded at a density of 100 cells per well. Colony-forming unit-fibroblast (CFU–F) was stained with Giemsa solution and counted by ImageJ.

### 2.7. Flow cytometry

The attached cells from passage culture were detached by trypsin digestion, washed in PBS, and reacted with FITC-conjugated or PE-conjugated monoclonal antibodies (BD, United States) against human CD34, CD45, CD73, CD90, and CD105 for 30 min in the dark at room temperature. The cells were washed twice in PBS, and at least 10000 events were collected with FACSCanto (BD, United States). The data were analyzed with FlowJo software.

### 2.8. The treatment of RGE to cultured UC-MSCs

The UC-MSCs at passage 3 were treated with RGE for primary assays, including *in vitro* differentiation assay and assay to determine the induction of the proliferation - migration of UC-MSCs.

This treatment divided the UC-MSCs population into three groups by various concentrations of RGE such as 50 ppm, 100 ppm, 150 ppm, respectively, R1, R2, R3 groups.

### 2.9. Cytotoxicity assay

Cell cytotoxic assay was performed for testing the cytotoxicity of the RGE against UC-MSCs. Initially, UC-MSCs were re-suspended with the fresh medium and counted. After that, UC-MSCs were seeded on a 96-well plate at the density of 10,000 cells/well and incubated in a CO_2_ incubator with 5% CO_2_ at 37 °C for 24 h for growth.

The viability rate of UC-MSCs was evaluated through Trypan blue assay like afore-research [20]. After the above incubation, the culture medium was removed, and these cells were exposed to serial 2-fold dilution of RGE (50 ppm, 100 ppm, 200 ppm, 400 ppm, 800 ppm, 1600 ppm, 3200 ppm, 6400 ppm) for 24 h. After treatment, the treated and untreated cells were stained with a previous procedure [21]. Briefly, these cells were incubated with 0.4% trypan blue for 10 min at room temperature (RT) after three washing times. These cells were fixed with 4% PFA solution and incubated at RT. Subsequently, the rate of viability cells was obtained by manually counting from the microscopic photos captured.

Finally, the online software named Quest GraphTM IC50 Calculator (AAT Bioquest, Inc., Sunnyvale, CA, USA) [22], was used for calculating the IC50 values for UC-MSCs after treatment, such as other research [20,23].

### 2.10. Proliferation assay by automatically counted using ImageJ

For proliferation analysis, UC-MSCs were passage cultured in StemMACS™ MSC Expansion Media Kit XF (Miltenyi Biotec, Germany). Cells were visualized using an Olympus CKX31SF inverted microscope (4× objective lens) and images were captured (three fields of view per replicate; three replicates). Cell number was assessed and compared using automated cell identification methods. This method already is demonstrated that it was sensitive enough to accurately detect both more minor changes in cell numbers and a more comprehensive range of cellular densities than spectrophotometric analysis of crystal violet-stained cells [24]. According to actual conditions, afore macro was changed:


//Convert Image to 8-bit
run("8-bit");
//Remove Noise
run("Despeckle");
//Adjust Brightness and Contrast
setMinAndMax(241, 255);
run("Apply LUT");
//Apply Phansalkar Local Threshold
run("Auto Local Threshold...", "method=Phansalkar radius=15 parameter_1=0
parameter_2=0 white");
setAutoThreshold("Default");
//Watershed
run ("Watershed");
//Count Objects (i.e. Cells)
run("Analyze Particles...", "display clear summarize");


### 2.11. Migration analysis by ImageJ based on scratch assay

For migration analysis, UC-MSCs were passage cultured to gain 70% confluence before a scratch assay was performed as described by previous research [25]. Briefly, the scratches were made with a sterile 5000-μl loading tip to create a linear ‘wound’ devoid of cells. The cells were then washed twice with sterile PBS, and fresh media was added. The cells were incubated for 24 h and images captured every 3 h by Olympus CKX30 microscope (4× objective lens; two fields of view per replicate, for two replicates). The captured image was analyzed with afore macro [24] using ImageJ software to carry out automated wound area measurements.

### 2.12. Demonstration regarding the cardiogenic–differentiation induction ability of RGE

UC-MSCs population after induction was evaluated with similar assays to our previous research paper [26], including investigation regarding shape index, the orientation of cell population, RT-PCR, and immunocytochemistry.

#### 2.12.1. Shape index analysis

Briefly, the shape index can measure cellular morphology [27]. This index was calculated in ImageJ by using the following equation and obtained through microscopic images taken on 0, 5, 10, 15 days after treatment. Every experiment was triplicated.

Equation:


$$\text{Shape index}=4\Pi \times \frac{Area}{{\left(Perimeter\right)}^{\hat{2}}}$$

#### 2.12.2. Analysis of the orientation of UC-MSCs population

ImageJ software was used to figure out the proportion of cells that were directed to alignment and orientation through θ angle [28,29]. This angle is formed by a vector of cells and vertical line (Fig. 4 L), and it ranges from 0° to 90°.

#### 2.12.3. Semi-quantitative RT-PCR

The total RNA extraction was performed using InviTrap® Spin Universal RNA Mini Kit (STRATEC Biomedical AG, Berlin), according to the manufacturer's instructions. Reverse transcription was reacted by Promega GoScript (TM) Reverse Transcriptase (Promega Corporation) and random hexamer primers to form first-strand with RNA templates. cDNA was diluted with Nuclease free water. Subsequently, PCR reactions used PHUSA Taq_500 and specific primers (Table 1). These primers were designed online using primer-BLAST (www.ncbi.nlm.nih.gov/tools/primer-blast/). All reagents and primers were purchased from PHUSA Biochem Company, Vietnam.

#### 2.12.4. Immunohistochemistry

Initially, UC-MSCs were fixed with methanol for 10 min at −20 °C, washed three times with PBS, followed by incubating at 4 °C. Primary antibodies, which were incubated with the cell for 1.5h at 37 °C, were diluted before (1:100). The next step was 30 min incubation with secondary antibody (diluted 1:200). Slides were taken photo by using diaminobenzidine substrate and counterstaining with hematoxylin.

#### 2.13. Statistical analysis

All data is shown as mean +standard deviation (SD), and differences between samples were determined by Student's t-test. One-way ANOVA, Turkey's HSD post-test among selected pairs of groups were also analyzed and performed with R software for Mac OS. Values with a p < 0.05, p < 0.01, p < 0.001 were considered statistically significant.

## 3. Results

### 3.1. Carbohydrate content of RGE

**Fig. 2:**
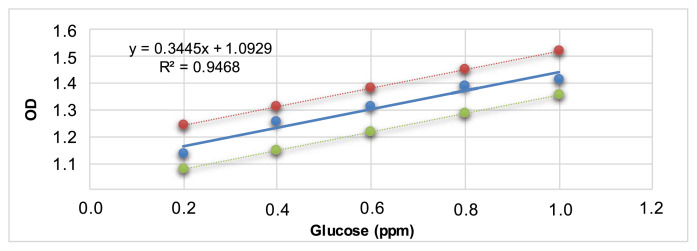
Standard curve with 95% prediction interval of absorbance at 490 nm on “Y” axis representing absorbance at 490 nm versus concentration of glucose in μg/mL (ppm) on X axis.

Data from the phenol - sulfuric acid method was used for making a standard linear curve by glucose content. Forward, carbohydrate concentrations in the samples were calculated based on the standard curve (Fig. 2).

From the inferred carbohydrate content given in Table 2, the carbohydrate content contained in 0.2; 0.4; 0.6; 0.8 μg/mL is 0.14; 0.29; 0.43; 0.60 μg/mL, respectively. Since carbohydrate is the major component in RGE, it can play the main role in many biofunctions of RGE. The RGE in this research has a similar carbohydrate content to previous research [7] and is suitable to test in the next assays.

### 3.2. Phytochemical analysis by GC–MS

**Fig. 3:**
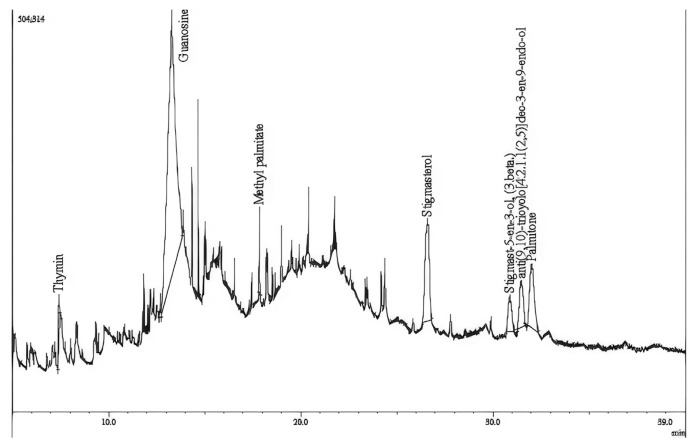
GC–MS chromatogram of hot water-extracted Rehmannia glutinosa.

The GC–MS spectrum was presented in Fig. 3, confirmed the presence of various components. This result was used for predicting the formula and structure of 7 biomolecules and their bioactives according to other publications, revealing various biological functions of different compounds in our extract (Table 3).

Several bioactive compounds from these plants have a specific role as bioactive mediators in regulating the rate of cell division and differentiation. In these compounds, most of them resemble afore-research, which is guanosine [30], methyl palmitate [31], palmitone [31], stigmasterol [32,33], and Anti (9,10)-tricyclo [4.2.1.1 (2,5)]dec-3-en-9-endo-ol was announced that present in Lactuca runcinata DC and never been seen in RG before [34].

### 3.3. UC-MSC characteristic evaluation

#### 3.3.1. MSC morphology

**Fig. 4:**
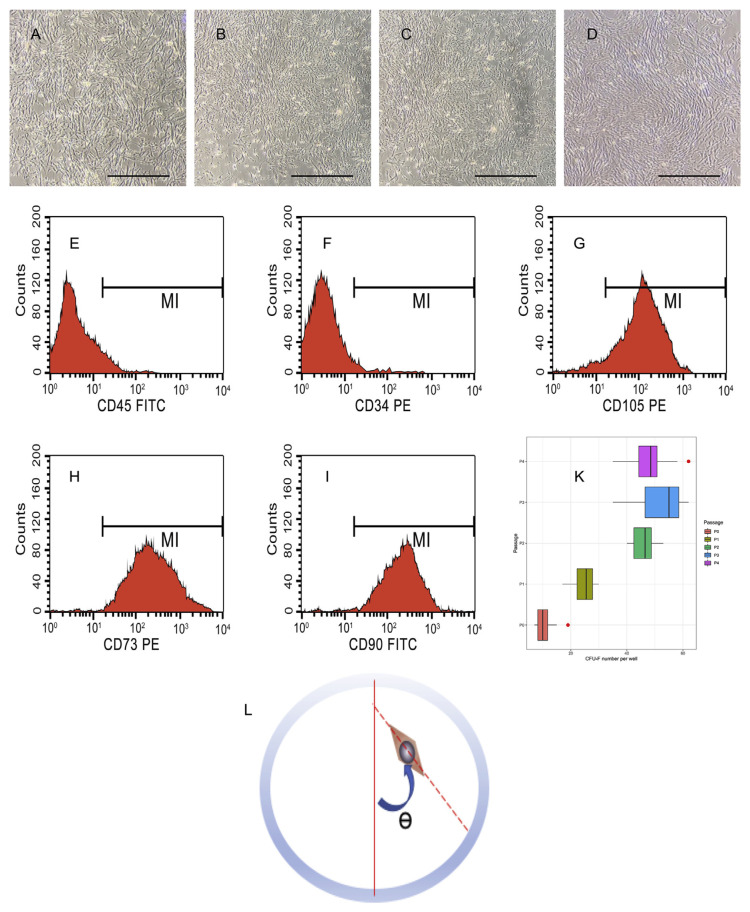
Evaluation of isolated UC-MSCs population (A–D) Fibroblast-like morphology of UC-MSCs at primary culture, passage 1 to 3, respectively. (E–F) Histograms of FACs were analyzed by FlowJo revealed that the UC-MSCs be negative for CD45, CD34 and positive for CD90, CD105, CD73. Scale bar = 1000 μm. (K) Results of CFU-F assay from P0 to P4. (L) The θ angle for evaluating the orientation of cell populations.

The cultured UC-MSCs population displayed a typically homogeneous fibroblast-like morphology (Fig. 4 A–D). This cell population was evaluated through the surface marker of UC-MSCs by flow cytometry analysis, which revealed the positive for CD73, CD90, CD105, and negative for CD45 and CD34 (Fig. 4 E–I).

#### 3.3.2. CFU-F assay results

After 3–5 days of culture, cells begin to divide and form small colonies around the original cell. From 7 to 10 days, CFU-F clusters form with a cell count greater than 50 cells per colony and its diameter more than 1 mm. Then, staining with Giemsa and counting with ImageJ software. The number of colonies constantly increased from the primary culture to the fourth passage (Fig. 4 K).

### 3.4. Cytotoxicity of RGE on UC-MSCs

**Fig. 5:**
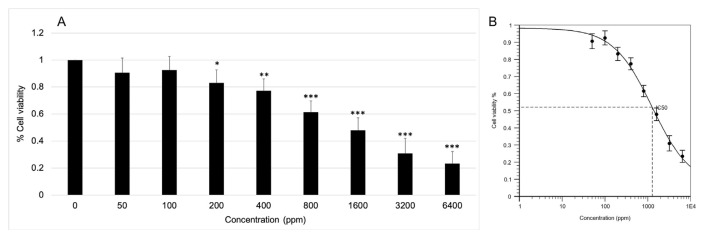
Results of cytotoxicity assays. (A) Percentage of cell viability with various concentrations of RGE. (B) IC50 result was performed by Quest GraphTM IC50 Calculator.

From the result was given by Quest GraphTM IC50 Calculator (Fig. 5 B), the cytotoxicity effect of RGE against UC-MSCs was presented through half-maximal inhibitory concentration (IC50) value, which is 1274 ppm (Fig. 5 B). The IC50 value is a measure of a substance's ability to inhibit a particular biological or biochemical activity. In vitro, it represents the concentration of a substance or drug necessary for 50% inhibition [45]. As a result of the high IC50 value (1274 ppm), RGE's cytotoxicity on UC-MSCs can be categorized as virtually non-toxic (>500 ppm), similar to previous research on another substance [23].

The result about the rate of cell viability after treatment indicated the statistically significant difference between treated cell viability (cell group treated with RGE of concentration from 200 ppm to 6400 ppm) and cell viability in the control group (Fig. 5 A). We determined that the highest safety concentration of RGE on UC-MSCs is just over 100 ppm. Thus, the following assays were performed with three RGE concentrations: 50 ppm, 100 ppm, and 150 ppm.

### 3.5. Effect of RGE extract on the proliferation and migration of UC-MSCs

#### 3.5.1. The proliferation stimulation ability

**Fig. 6:**
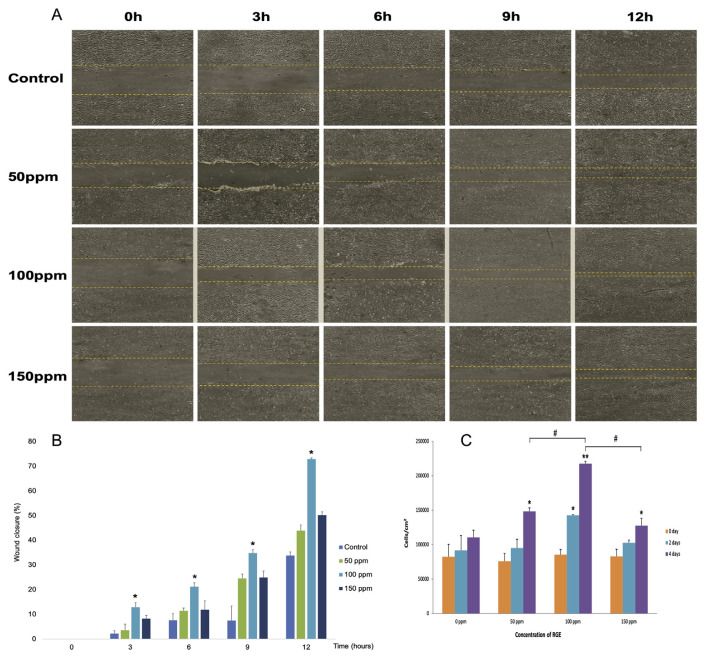
Results of RGE effect on the proliferation – migration of UC – MSCs (*, #p < 0.05; **< 0.01). (A) Microscopic photos of scratch model in vitro. (B) The area of wound of different group carried out by ImageJ software. (C) The number of cells of various groups during 4-day course.

The cell number was counted by ImageJ software revealed the power of RGE on proliferation stimulation by all three various concentrations (Fig. 6C). At the RGE concentration of 50 ppm and 150 ppm (group R1, R3, respectively), the proliferation promotion ability of RGE only exhibits on the sixth day after treatment, which is evaluated through the significant difference in the number of cells between the sixth day after treatment and before treatment (0 days). Besides, since the statistically significant difference in the number of cells appeared from the third day from treatment in the R2 group, the best RGE concentration for proliferation purpose is 100 ppm. Because of that difference in proliferation stimulation between various groups, the UC-MSCs number in the R2 group was higher than equivalent figures in R1 and R3 groups on the fourth day.

#### 3.5.2. Result of the scratch assay

The reduction of wound area when treating with RGE reflects this extract's ability to stimulate the migration and proliferation of UC-MSCs. Results this assay displayed through photomicrograph (Fig. 6A) and ImageJ-based analyzed results (Fig. 6B) indicated that speed of scratch healing in all treated groups was faster than the control group (0 ppm), especially the RGE possibility regarding the wound healing stimulation in the R2 group is more significant than rest groups, which is shown that after 12 h of treatment the wound closed around 80%. This result agrees with the above output that the RGE concentration of 100 ppm is the most suitable concentration for stimulating the proliferation of UC-MSCs.

**Fig. 7:**
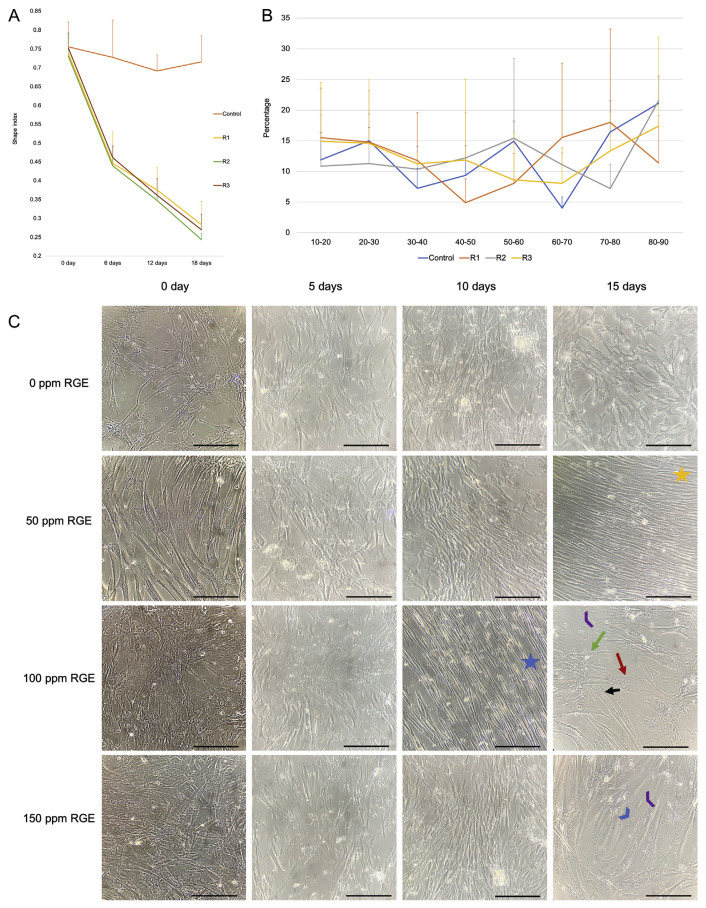
The changes in morphology of cells and orientation of cell population (Scale bar: 200 μm). (A) The change in shape index of the cells after treatment. (B) The alignment of the cell population after treatment. (C) The microscopic images of the shape of cells under treatment. (Star: the orientation; red arrow: myotube – like cells with two nuclei; black arrow: the junction of two cells; green arrow: gathering; purple arrowhead: cytoplasmic striations; blue arrowhead: two - nuclei.)

Initially, the ability of RGE to induce the cardiogenic pre-differentiation from UC-MSCs was exhibited through the morphological changes traced by shape index and the orientation of the cell population after treatment. This work revealed that the shape index of the treated UC-MSCs constantly falls in the period after treating with RGE, for details from around 0.75 at the 0 days to approximate 0.25 at the 18 day under treating (Fig. 7 A). This outcome similar to afore publishes [46](Dat Huu Nguyen et al., 2020), which announced the more elongation and spindle-shaped in the cardiogenic differentiation process. In all concentrations of RGE, it is clear that they are responsible for changing the shape index of UC-MSCs, which explained for adapt behavior of UC-MSCs to the bioactive RGE.

Besides the result, which was quantitated by ImageJ software, the morphology of UC-MSCs was also visualized and shown in Fig. 7C. In some different microscopic fields, we can find out some specific shapes and expressions. Some treated cells had two nuclei (red arrow, blue arrowhead). In contrast, others seemed elongated, so look like rods (star), with the appearance of cytoplasmic connecting processes between adjoining cells to form the myotube-like shapes (blue arrowhead) with the junction-like shape (black arrow). Other cells also were organized in gatherings (green arrow), and the majority of treated cells had a ton of cytoplasmic striations (purple arrowhead). Furthermore, these specific morphologies appeared spontaneously, and no significant difference between distinguished groups.

On the contrary, the orientation of the UC-MSCs population did not change during this assay. This result showed through the random distribution of θ angles from 10° to 90° of all cell groups Fig. 7 B. This outcome indicated no orientation effect of RGE on the whole treated cell population, despite the appearance of orientation in some local sites (blue star, orange star).

### 3.6. Expression of some molecular myocardial markers

#### 3.6.1. Semi-quantitative RT-PCR assay

**Fig. 8:**
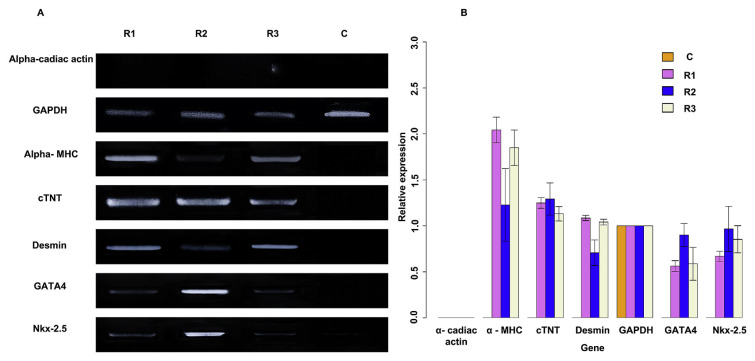
The results of RT-PCR assay. (A) Electrophoresis result of surveyed genes of various groups. (B) Quantity results were given by ImageJ software.

After performing the RT – PCR assay, the photos of electrophoresis were captured for analyzing these concerned bands (Fig. 8A). According to the result in Fig. 8, five out of six specific surveyed genes expressed, which are *GATA4, Nkx-2.5, cTNT, α- MHC, Desmin*. *GAPDH* was reference genes and used as internal reference controls for normalizing these bands for semi-quantitative by ImageJ software. The semi-quantitative analysis result revealed the expression of the five above genes and the silence of *α- cardiac actin* gene.

#### 3.6.2. Immunocytochemistry

**Fig. 9:**
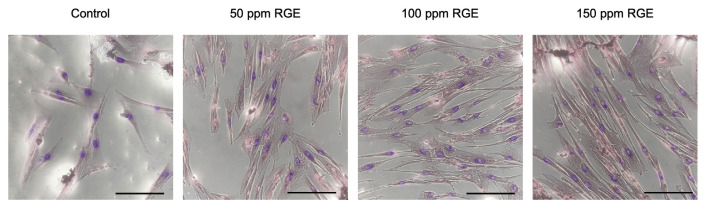
Immunocytochemistry assay for Desmin protein stain (Scale bar: 200 μm).

Following the verified upregulation of some cardiac-specific mRNA, the result of differentiation was confirmed on the specific protein expression. Due to our laboratory's condition, we proved the expression of Desmin by immunocytochemistry, which is a particular protein of cardiac muscle tissue. This assay indicated that the RGE-treated groups after treatment expressed Demin protein, while cells in the control group did not display Desmin (Fig. 9).

## 4. Discussion

Initially, these results regarding the tests on the phytochemicals of RGE revealed that RG is a carbohydrate-rich plant and has many different bioactivities that were predicted through the components in RGE obtained in GC-MSs result and other previous publications. Among these predicted effects pharmacologically, it is easy to guess that this extract has positive effects on the mesenchymal stem cells because MSCs play a prominent role in the recovery process of many diseases. Thus, unsurprisingly we discovered that RGE in this research could not have the cytotoxicity effect on the UC-MSCs population. This result about the cytotoxicity is contrary to a previous result that RGE can have cytotoxicity effect on a cancer cell line [7].

All the tested RGE concentrations not only are safe for the growth of UC-MSCs but also have proliferation – migration stimulation effect on the UC-MSCs. Significantly, the RGE concentration of 50 ppm displayed the most potent stimulation ability on proliferation and migration. These results were confirmed through the scratch assay and the cell counting assay, and these results are also similar to previous publish that RG can stimulate the proliferation of cells [47,48]. However, this ability is only showed during 4 days after treatment, and from the fifth day after treatment with RGE, the morphology of MSCs was changed, which is exhibited the early changes in cell type.

After that, for investigating the cardiogenic differentiation, the cell population after treatment was evaluated the morphology and the orientation of the whole population from the fifth day after treatment. It is clear that from the fifth day, the morphology of the cell has significant changes, which is become more elongated and turn to tubule-shaped from the fibroblast-shaped and spindle-shaped of MSCs, which were display through the photos and the reduction in the shape index and this results also is the same with many previous cardiogenic differentiation researches [49]. Besides, some cells after treatment also exhibited some specific morphologies of cardiomyocytes like two-nuclei, the display of disc-like shape through the connecting process of adjoining cells, the gathering, and the cytoplasmic striations similar to previous research of the cardiogenic differentiation effect of 5-aza [50].

Although the alignment is the main specific character of the mature heart muscle tissue, either this differentiation strategy or common differentiation strategy, which is 5-azacytidine use, had limited due to failure in making the alignment [46]. Besides, we had succeeded in generating aligned cardiomyocyte – like cells from UC-MSCs when using the electrical field for differentiation purposes [26]. Despite the RGE treated – cells were not express the alignment on the whole cell population or all the microscopic fields, the treated cells in some areas also align and display a clear orientation. Due to this local alignment or the orientation in the different areas was different from each other, the statistical figures for this trait (θ angle) in the whole cell population fluctuate greatly and generation the high standard deviation as above result.

From the results of PCR assay, it is evident that our UC-MSCs population do not express the *GATA4* and *Nkx-2*.5 mRNA, which is similar to other published data [51], while some conventional protocol can isolate the cell population with the appearance of *GATA4* and *Nkx-2*.5 mRNA at a low level [52]. This controversial result can come from the sources of MSCs as also as the isolation position. The expression of somemRNAthat plays role as myocardial markers was detected by PCR assay such as GATA4, *Nkx-2.5, cTNT, α- MHC, Desmin*. Among these, *GATA4* and *Nkx-2.5* are early markers and were shown to modulate specific genes during the early-stage development of cardiogenic differentiation [53]. Thus, the RGE also can be seen as is an epigenetic modulator; this might be from DNA demethylation of the CpG islands of the promoter regions of *Nkx2.5* and GATA4 clears the path to access these promoters for active transcription [54].

Besides, some mature myocardial markers like *cTnT, α- MHC* were also up-regulated in all three treated groups. The semi-quantitative results revealed the high expression of these markers compared to the expression of both *GAPDH* as control and other early markers. We also performed the PCR reaction to indicate the expression of *Desmin* mRNA during treatment. Unfortunately, in this work, although 5 out of 6 surveyed specific genes as either soon marker or later marker upregulated under RGE treatment, the last later marker is *α- cardiac- actin* was not expressed at any cell groups. This lack of expression can bring surprise due to the *α- cardiac- actin* one primary marker responsible for gathering muscle proteins and coordinating contractile reaction in the mature heart muscle tissue. This gene's silence can prove the failure in differentiation into functional cardiomyocytes from UC-MSCs by using bioactive compounds form RG [8]. However, the exhibit of some markers like *cTnT, α- MHC, GATA4, Nkx-2.5, Desmin* could be confirmations of differentiation into cardiomyocyte-like cells [55].

## 5. Conclusion

The hot water-extracted *R. glutinosa* Libosch in this research has a large proportion of carbohydrate contents and has diverse pharmacological effects are predicted from the result of GC–MS. This extract is categorized as non-toxic to UC – MSCs with IC50 value is 1274 ppm. All the tested concentrations of RGE can stimulate the proliferation–migration process during the initial four days of treatment and induce the cardiogenic differentiation from the fifth day of treatment, which is demonstrated through the reduction in shape index, some specific morphology (gathering, cytoplasmic striations, two-nuclei), the local orientation, the expression of specific genes (*GATA4, Nkx- 2.5, cTnT α- MHC, Desmin*), and the expression of Desmin protein.

## Figures and Tables

**Table 1:** Primers are designed and used in RT-PCR assay.

Primer	Sequence	NCBI reference gene
GAPDH_F	CAG GGC TGC TTT TAA CTC TGG	NM_001289746
GAPDH_R	AGG GAT CTC GCT CCT GG	
cTNT_F	ATG AAG ATC AGC TGA GGG AGA A	NM_001276347
cTNT_R	GTC GAA CTT CTC TGC CTC CAA G	
Desmin_F	TGC CCT CAA CTT CCG AGA AAC	NM_001927
Desmin_R	ACT TCA TGC TGC TGC TGT GT	
Nkx-2.5_F	GAG CCG AAA AGA AAG CCT GAA A	NM_001166175
Nkx-2.5_R	TCC CTA CCA GGC TCG GAT AC	
GATA-4_F	CCG TGT CCC AGA CGT TCT C	NM_001308093
GATA-4_R	GCA TAG CCT TGT GGG GAG AG	
α-MHC _F	TCC TGC GGC CCA GAT TCT TC	NM_002471
α-MHC _R	TCC GGA CAG TCT TGG CAT TG	
Alpha cardiac actin_F	TAT GCT TCT GGC CGT ACC AC	NM_005159
Alpha cardiac actin_R	GTT GCA AGT CCT GGT CTG GT	

**Table 2:** The absorbance of RGE samples at different concentrations infer its carbohydrade content.

Concentration of RGE (μg/mL)	Absorbance	Carbohydrate content (μg/mL)
0.2	1.142	0.14
0.4	1.194	0.29
0.6	1.241	0.43
0.8	1.301	0.60

**Table 3:** The components of RGE and their predicted bioactivities.

Name of compound	Peak area (%)	Bioactives
Thymin	1.81	Activities against *Bacillus pumilus*, *Proteus vulgaris*, and *Escherichia coli*. [35]
Guanosine	64.43	Neuroprotective effects through the reduction in apoptosis, reduction in glutamate toxicity, induction of hemoxygenase-1 (HO-1), … [36]
		Generation of guanosine monophosphate (GMP), cyclic guanosine monophosphate (cGMP), guanosine diphosphate (GDP), and guanosine triphosphate (GTP) through phosphorylated reaction
Methyl palmitate	1.56	Cardioprotective activities through antioxidant, anti-inflammatory, anti-apoptotic, anti-fibrotic [37]
Stigmasterol	13.19	Apoptotic inducement in HepG2 cells [38]
		Proliferative inhibition in smooth muscle cell [39]
		Immunomodulation [40]
		Stigmast-5-en-3-ol, (3.beta.) 3,96 Anti-diabetic potency [41]
		Apoptotic and antiproliferative effects on human breast cancer cells [42]
Anti (9,10)-tricyclo [4.2.1.1 (2,5)]dec-3-en-9-endo-ol	5.8	Contribution for bioactive of *Lactuca runcinata* DC [34]
Palmitone	9.26	Inhibition on human ovarian cancer cell without any cytotoxic on human PBMCs [43]
		Antibacterial activity against *Streptococcus viridans*, *Staphylococcus aureus*, *Staphylococcus albus*, *Escherichia coli*, *Pseudomonas pyocyanea*, *Bacillus subtilis*, *Pseudomonas aeruginosa*, *Klebsiella aerogenes*, *Bacillus sphaericus*, *Chromobacterium violaceum* [43,44]
		Antifungal activity against *Aspergillus niger*, *Rhizopus oryzae*, *Beauveria bassiana*, *Fusarium moniliforme*, and *Curvularia lunata* [44]
